# Preterm birth leads to a decreased number of differentiated podocytes and accelerated podocyte differentiation

**DOI:** 10.3389/fcell.2023.1142929

**Published:** 2023-03-02

**Authors:** Lulu Zhang, Zhihui Chen, Qi Gao, Ge Liu, Jun Zheng, Fangrui Ding

**Affiliations:** ^1^ Department of Neonatology, Tianjin Central Hospital of Obstetrics and Gynecology, Tianjin, China; ^2^ Tianjin Key Laboratory of Human Development and Reproductive Regulation, Tianjin, China; ^3^ Department of Neonatology, Nankai University Maternity Hospital, Tianjin, China

**Keywords:** preterm, podocyte, chronic kidney disease, single cell sequence (scRNA-seq), ribosome

## Abstract

Preterm birth was previously identified as a high-risk factor for the long-term development of chronic kidney disease. However, the detailed pattern of podocyte (PD) changes caused by preterm birth and the potential mechanism underlying this process have not been well clarified. In present study, a rat model of preterm birth was established by delivery of pups 2 days early and podometric methods were applied to identify the changes in PDs number caused by preterm birth. In addition, single-cell RNA sequencing (scRNA-seq) and subsequent bioinformatic analysis were performed in the preterm rat kidney to explore the possible mechanism caused by preterm birth. As results, when the kidney completely finished nephrogenesis at the age of 3 weeks, a reduction in the total number of differentiated PDs in kidney sections was detected. In addition, 20 distinct clusters and 12 different cell types were identified after scRNA-seq in preterm rats (postnatal day 2) and full-term rats (postnatal day 0). The numbers of PDs and most types of inherent kidney cells were decreased in the preterm birth model. In addition, 177 genes were upregulated while 82 genes were downregulated in the PDs of full-term rats compared with those of preterm rats. Further functional GO analysis revealed that ribosome-related genes were enriched in PDs from full-term rats, and kidney development-related genes were enriched in PDs from preterm rats. Moreover, known PD-specific and PD precursor genes were highly expressed in PDs from preterm rats, and pseudotemporal analysis showed that PDs were present earlier in preterm rats than in full-term rats. In conclusion, the present study showed that preterm birth could cause a reduction in the number of differentiated PDs and accelerate the differentiation of PDs.

## Introduction

Preterm birth was previously identified as a high-risk factor for the development of long-term chronic kidney disease (CKD) (Hoy et al., 1999; Crump et al., 2019; Ding et al., 2021a; Gao et al., 2022). The most widely accepted mechanism by which preterm birth could increase the risk of developing long-term CKD is the “nephron number hypothesis”, which holds that preterm infants or low-birth-weight infants who are born with fewer nephrons have an increased risk for renal disease later in life (Brenner et al., 1988; Luyckx et al., 2013; Luyckx, 2017; Perico et al., 2018). Both human and animal studies have confirmed that premature birth has adverse effects on kidney development and nephron numbers (Rodriguez et al., 2004; Gubhaju and Black, 2005; Sutherland et al., 2011; Stelloh et al., 2012; Ryan et al., 2018). In animal studies, a preterm mouse model in which pups were delivered 1 or 2 days early by cesarean delivery was established, and after a few weeks of feeding, the glomeruli were counted. Fewer nephrons were present in preterm mice than in full-term animals (Stelloh et al., 2012). This study directly confirmed the effect of preterm birth on nephron numbers. In human studies, although there is little direct evidence concerning nephron numbers in the context of preterm birth, researchers have examined kidney tissue collected at autopsy from preterm neonates and showed that preterm kidneys had a greater percentage of morphologically abnormal glomeruli, which suggested that the preterm kidney may have fewer functional nephrons, thereby increasing vulnerability to impaired renal function both in the early postnatal period and later in life (Sutherland et al., 2011; Ryan et al., 2018).

In addition to the nephron number hypothesis, in our recent study, we put forward the involvement of podocyte (PD) depletion hypothesis during this process (Ding et al., 2021a; Gao et al., 2022), which holds that preterm birth accelerates PD depletion during growth and in turn contributes to a high risk for future CKD development. In our human study, a more than 5-fold increase in urinary PD excretion was detected in preterm infants when the corrected gestational age matched that of full-term infants (Gao et al., 2022). In an animal study, higher urine PD excretion was detected at the cross-section time point, and persistently higher PD depletion was detected during long-term follow-up until the age of 12 months in rats, which corresponded to middle adulthood in humans. More importantly, gradually accelerated PD depletion was observed during this follow-up. The same finding was also confirmed in kidney biopsy samples in an animal study (Ding et al., 2021a). According to the PD depletion hypothesis, if progressive PD depletion occurs over time, it leads to proteinuria, progressive glomerulosclerosis and progressive loss of renal function culminating in end-stage kidney disease (Kim et al., 2001; Kriz, 2002; Wiggins, 2007; Ding et al., 2017). Thus, our previous studies confirmed the important role of PD and PD depletion in the risk of developing CKD after preterm birth.

During normal kidney development, nephron development begins in the 9th gestational week, with rapid proliferation in the last trimester, and ceases in approximately the 36th week (Brenner and Milford, 1993; Hoy et al., 2005; Faa et al., 2010; Ryan et al., 2018). Most preterm infants are born from 21 to 37 gestational weeks, corresponding to the second and third trimesters, which indicates that prematurity interrupts the normal physiological development of the kidneys (Patel et al., 2015; Stoll et al., 2015). Compared with full-term kidney, the preterm kidney begins performing functions such as waste filtration independently very early after birth, while most work of the full-term kidney is done by the maternal placenta. Thus, the development of the preterm kidney includes two stages: the intrauterine stage before preterm birth and the abnormal extrauterine stage. In full-term infants, the kidneys experience only the intrauterine stage before birth. The major difference between the preterm and full-term kidney is that the preterm kidney experiences an extrauterine stage of independent work. This abnormal stage may have an effect on the development and differentiation of PDs and subsequent outcomes. To thoroughly understand and investigate the possible mechanism during this process, in the present study, a preterm rat model was established by delivery of pups 2 days early. After 2 days, when the preterm rats had experienced the extrauterine stage of independent work for 2 days, the kidneys from these preterm rats and from full-term rat pups were collected. Then, single-cell RNA sequencing (scRNA-seq) and subsequent bioinformatic analysis were performed. The aim of the current study was to provide a new understanding of the mechanism of long-term CKD caused by preterm birth.

## Materials and methods

### Animals

This study was approved by the Nankai University Animal Research Ethics Committee (No. 2021-SYDWLL-000148). The use and care of experimental animals as well as all experimental protocols followed the Regulations for Experimental Animal Use issued by the State Committee of Science and Technology of the People’s Republic of China and ARRIVE guidelines. Adult male and female Sprague–Dawley rats were purchased from Charles River Laboratories (Beijing, China) and then caged together at a 1:1 ratio. Pregnant rats were confirmed both by the vaginal plug and significant enlargement of the abdomen days after vaginal plug presentation. For preterm rats, cesarean delivery was performed at 20 days of gestational age. Then, the pups were collected and distributed to female rats who had delivered within 3 days for suckling. Full-term rats were obtained *via* vaginal spontaneous labor at 22 days of gestational age. In podometric experiments, male rats in both the preterm and full-term groups were sacrificed for the collection of kidney samples at the age of 3 weeks. In single-cell sequencing experiments, preterm pups were sacrificed at 2 days post-birth, and full-term rats were sacrificed after birth for collection of kidney samples. All rats (pups) were anesthetized by CO_2_ inhalation.

### Podometric processing of kidney samples

This method was performed as previously described (Venkatareddy et al., 2014; Ding et al., 2017) and is detailed as follow. After samples collection, kidneys were divided equally into two parts in cross section and then embedded in paraffin. Three-micron-thick paraffin sections were placed on slides for staining. During immunostaining, slides were deparaffinized in fresh xylene, rehydrated and then permeabilized in Triton X-100. Then, antigen retrieval was performed. After blocking with BSA for 2 h, primary antibody (WT1 SC-7385, Santa Cruz) diluted 1:50 in 1% BSA was incubated with the slides overnight at 4°C. Then, the slides were incubated with the secondary antibody, Alexa Fluor 488 goat anti-mouse IgG (A11001; Invitrogen), diluted 1:300 and the tertiary antibody, Alexa Fluor 488 donkey anti-goat IgG (A11055; Invitrogen), diluted 1:300 in 10% human serum in PBS at room temperature for 2 h. The nuclei were stained, and the slides were mounted by using SlowFade Gold antifade reagent with diamidino-2-phenylindole (S36939; Invitrogen). The glomeruli were imaged by using a fluorescence microscope (Leica DMI8). During this process, more than 30 consecutive glomeruli were imaged with the “Z” method, and images were collected for further analysis. During this immunostaining process, Alexa Fluor 488-conjugated secondary and tertiary antibodies with a green signal were used. When further analyzing these images, images were acquired with an RGB filter, and the green signal was converted to red and *vice versa* (this method was developed by Roger Wiggins’ lab (Venkatareddy et al., 2014; Yang et al., 2015)). After image collection, the coverslips were removed, and the slides were processed for immunohistochemical staining according to PK-6102 (Vector). The slides were blocked in horse serum overnight at 4°C and then incubated with the primary antibody against synaptopodin (SYNPO) diluted 1:500 (65294, Progen) for 2 h at room temperature. After incubation with the secondary antibody from the kit, the substrate diaminobenzidine (D4293 Sigma) was used to develop the brown peroxidase product. Then, the slides were counterstained with hematoxylin, dehydrated, and mounted with resin. Finally, the same glomeruli were imaged by microscopy (Olympus DP72).

For PD nuclear density, PD nuclear number (WT-1) in each glomerulus, the apparent caliper diameter of PD nuclei and the apparent glomerular area were measured by using Image-Pro Plus 6.0 software. After performing this measurement on consecutive glomeruli captured in each sample, the mean apparent PD nuclear number, the mean diameter of PD nuclei and the mean glomerular area were determined. Then, the PD density of each sample was corrected by the method established by Venkatareddy et al. (Venkatareddy et al., 2014), and the PD density was determined. For the PD-positive area, the area of SYNPO positivity was measured and then calculated by the percentage of glomerular area.

### Single-cell isolation

The intact kidneys were collected from both preterm and full-term pups and then minced into millimeter-sized pieces in DMEM/F12 (Gibco A4192002) containing 10% FBS (Gibco 10099141C). Then, the tissues were transferred to digestion solution, DMEM/F12 containing 1 mg/mL Collagenase type II (Sigma C6885) and 100 U/mL DNase I (Roche 4716728001), at 37°C with agitation for 15–20 min. During this process, samples were pipetted up and down every 5 min, and 20 µL samples were used to confirm the status of digestion. After digestion, the samples were filtered through a 40-mm nylon cell strainer (BD Falcon), and then the filtered liquid was centrifuged at 800 rpm for 5 min. After removal of the supernatant, the pellets were washed three times with DPBS and finally resuspended by adding an appropriate volume of DPBS.

### Generation and sequencing of the 10x genomics RNA library

After collection, the single-cell suspension was input into a 10X Genomics Chromium device to capture 6,000–8,000 cells and generate single-cell gel beads in emulsions (GEMs) according to the manufacturer’s instructions for the 10X Genomics system. After the generation of GEMs, reverse transcription reactions were performed, and then barcoded cDNA was purified with Dynabeads. Then, a unique molecular identifier (UMI) was added to identify PCR duplicates. Thereafter, the samples were incubated with appropriate enzymes to produce full-length cDNA, and PCR amplification was conducted to generate sufficient quantities of material for library construction. The cDNA libraries were constructed and sequenced by the NovaSeq platform (Illumina) to generate 150 bp paired-end reads according to the manufacturer’s instructions.

### Data processing and analyses

After sequencing data were generated, Cell Ranger (version 3.1.0) was used to process the raw data. As a quality control step, cells with fewer than 500 unique expressed genes were excluded. In addition, cells with more than 6,500 genes were considered cell duplicates and excluded. Cells were also discarded if their mitochondrial gene percentages were over 30%. Moreover, the batch effects on single-cell RNA-sequencing data were removed by Harmony. Then, 32624 genes from 13815 cells were selected for further analysis. The gene expression matrix was normalized and log transformed by NormalizedData function. We identified 2000 highly variable features by FindVariableFeatures function. Using the variable genes as input, principal component analysis (PCA) was performed to reduce data dimension. After that, 13815 cells were clustered into 20 clusters by TSNE. In addition, differentially expressed genes (DEGs) were identified for each cell type with FindMaker function. In pseudotime analysis, the Monocle2 (version 2.10.0) algorithm was used to determine the translational relationships among cell types. GO and KEGG analyses were performed by applying the clusterProfiler package (v3.10.0).

### Statistical analysis

The Wilcoxon rank-sum test was used to compare the PD nuclear density and area density between preterm and full term rats. In addition, the chi square test was used to compare the proportion of each cell type between preterm and full term groups. A *p*-value <0.05 was considered statistically significant.

## Results

### Reduction in the number of differentiated mature PDs caused by preterm birth

**FIGURE 1:**
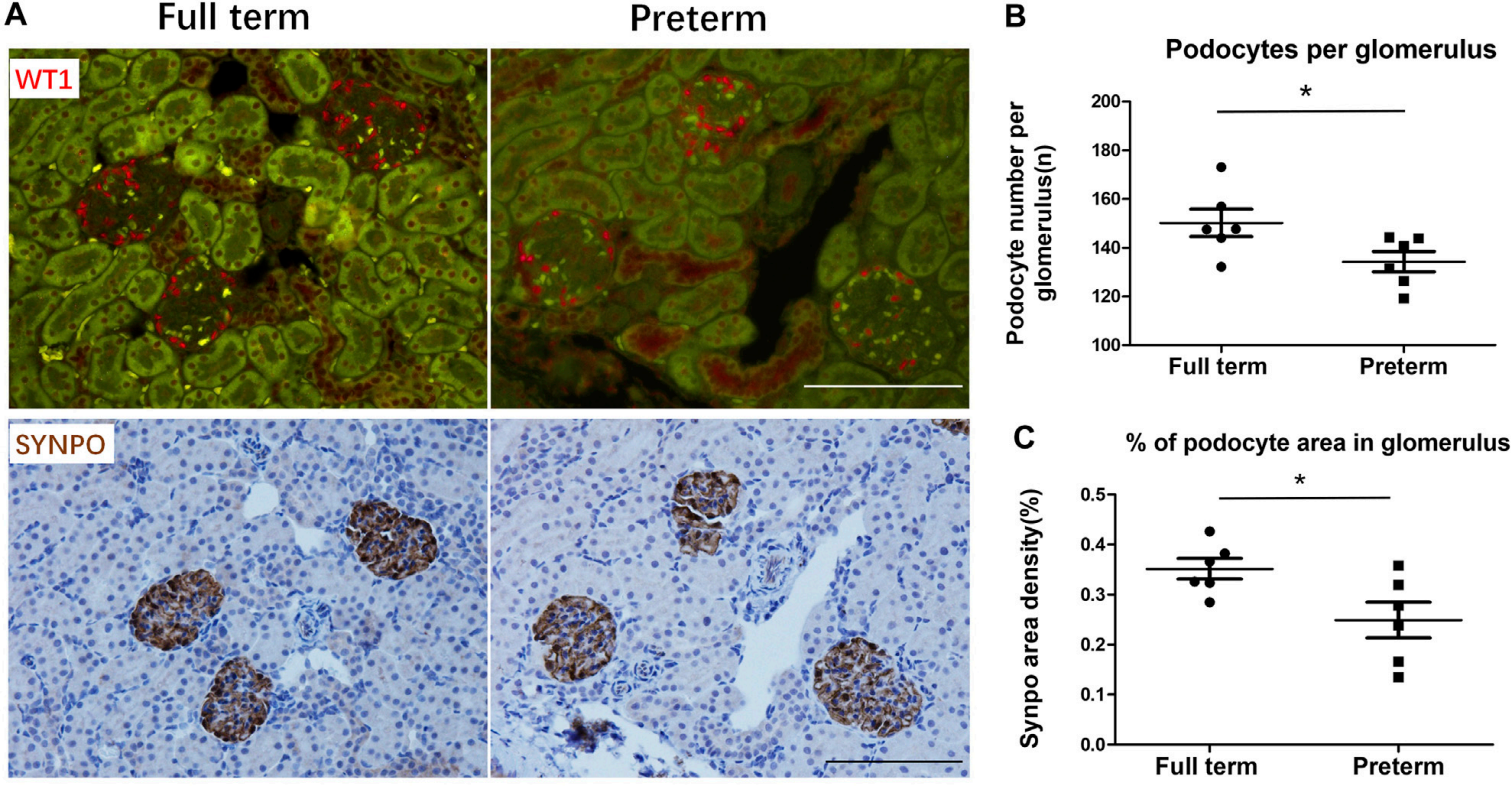
Preterm birth causes a reduction in the number of differentiated podocytes (PDs) at 3 weeks postnatally **(A)** The upper panels show WT1-stained PD nuclei (red). The lower panels show SYNPO peroxidase-stained PD cell bodies (brown) in the same sections. The left panels show full-term rat glomeruli. The right panels show preterm rat glomeruli. Bar = 100 µm. **(B,C)** At 3 weeks postnatally, PDs have already finished differentiating. The podometric parameters of PD nuclei per glomerulus and percentage of PD area in the glomerulus showed that compared with full-term rats (n = 6), preterm rats (n = 6) had significantly decreased PD numbers (*p* < 0.05) and PD area (*p* < 0.05). (T tests were used to compare parameters between full-term and preterm rats, *: *p* < 0.05).

When the kidney completely finished nephrogenesis at the age of 3 weeks, the PD number was estimated by measuring PD-specific nuclear protein WT-1-positive nuclei per glomerulus, and the PD area was estimated by measuring the PD-specific protein synapopodin area as x% of the glomerular tuft area. As shown in Figures 1A, B, in preterm rats, the PD nuclear number per glomerulus was significantly decreased compared with that in full-term rats. The PD area density was also significantly reduced in preterm rats compared with full-term rats (Figures 1A, C). Therefore, preterm birth results in a reduction in the number of differentiated PDs.

### Single-cell transcriptomic sequencing and clustering of kidney cells from age-matched preterm and full-term rats

**FIGURE 2:**
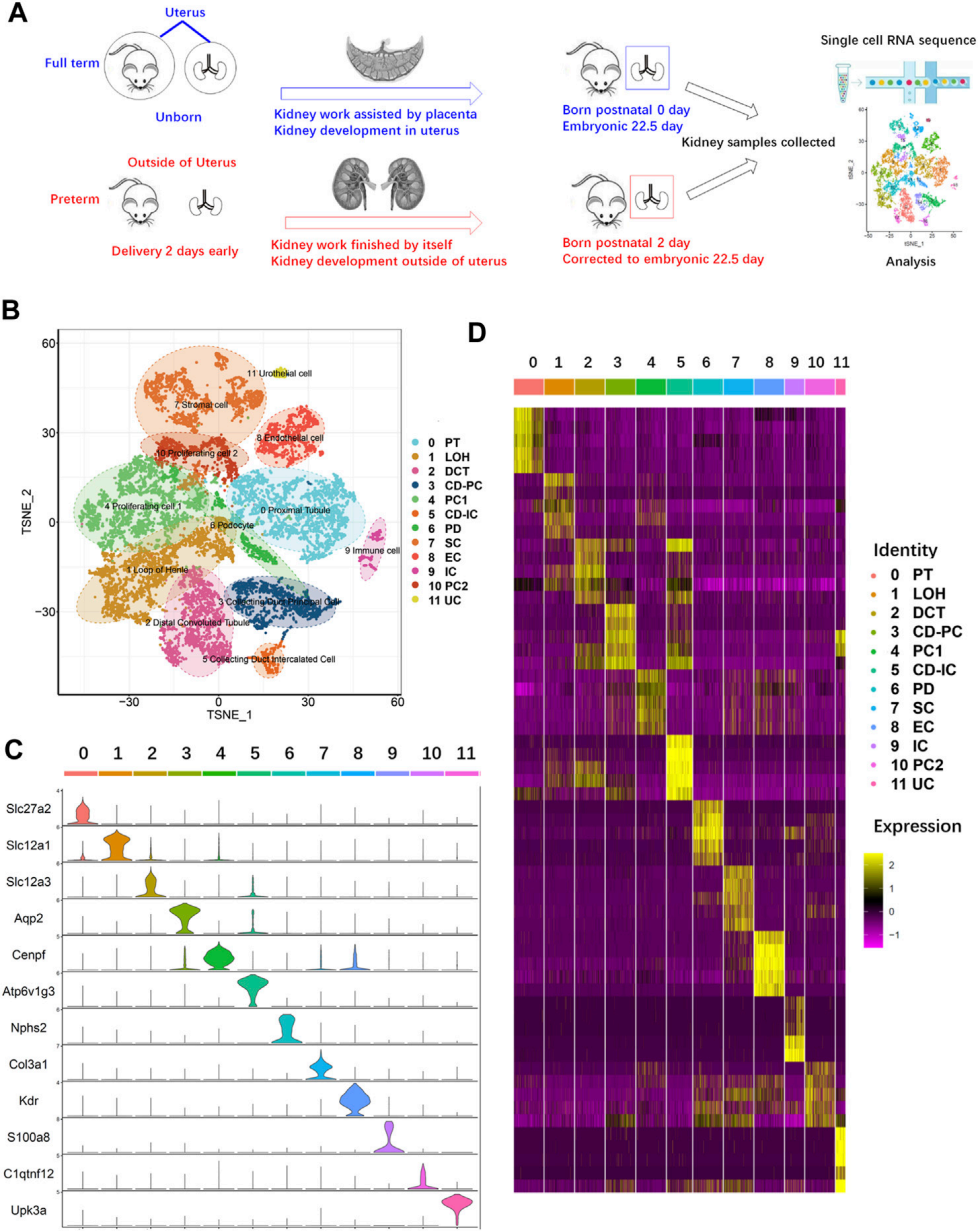
Single-cell RNA sequencing of kidney cells from age-matched preterm (postnatal day 2) and full-term rats (postnatal day 0). **(A)** Overview of the study design and experimental procedure. **(B)** Full-term and preterm rat kidneys were collected at birth and 2 days postnatally, respectively. After single-cell suspensions were generated, sequencing and analysis were performed. A total of 13815 cells and 20 original clusters were identified. After analysis of differentially expressed genes in the 20 original clusters with known gene markers, 12 different cell types were identified and annotated on the right side of the TSNE results. **(C)** Violin plots showing the expression levels of representative marker genes across the 12 clusters. The vertical axis shows the gene expression value. Each color represents one cluster, as indicated on the right side in **(D)**. The annotations of each color are presented on the right side of **(D)**. **(D)** Heatmap of the gene expression patterns of the top ten cluster-specific genes in 12 clusters. Different clusters are identified by different colors, as indicated at the top of the heatmap and annotated on the right side of the heatmap. The color bar ranging from yellow to red–purple reflects the relative expression levels from high to low.

To explore the possible mechanism of the abnormal changes in PDs caused by preterm birth, the major difference between preterm and full-term rats was identified and is shown in Figure 2A. As speculated, the difference in kidney development between preterm and full-term rats is that preterm kidneys experience an abnormal developmental stage, namely, the extrauterine stage, while full-term kidneys develop intrauterine. To explore the changes caused by abnormal kidney development in preterm rats, kidneys were collected from 2-day-old preterm rats and age-matched full-term rats at birth (Figure 2A). The kidneys in preterm rats have already worked independently for 2 days. The work of kidneys in full-term rats is assisted by the placenta. Both kidneys were collected from rats in the two groups, and single-cell RNA sequencing was performed. Finally, 13815 cells were obtained (6,812 cells from full-term rats and 7,003 cells from preterm rats). Further analysis yielded 20 original clusters (the median expression levels of each gene in each cluster are provided in Supplemental Table S1). After annotating DEGs by known gene markers of different kidney cell types, 12 different kidney cell types were identified overall (Figures 2B–D).

As shown in Figures 2B, C, cluster 0 represented the proximal tubule (PT) with high expression of the PT-specific gene Slc27a2 (Khan et al., 2018). Cluster 1 highly expressed Slc12a1 and belonged to the loop of Henle (LOH) (Castrop and Schnermann, 2008). Cluster 2 represented the distal convoluted tubule (DCT), with high expression of Slc12a3 (Syrén et al., 2002). Cluster 3 highly expressed the principal cell of the collecting duct (CD-PC) marker gene Aqp2 (Sabolić et al., 1995). In cluster 4, the proliferating cell 1 (PC1) marker gene Cenpf was highly expressed (Lindstrom et al., 2018a). For identification of intercalated cells of the collecting duct (CD-ICs), Atpv1g3 was selected, and cluster 5 was identified (Park et al., 2018). PDs highly express Nphs2, and cluster 6 was identified (Boute et al., 2000). Stromal cells (SCs) highly express Col3a1, which was identified in cluster 7 (Yamazaki et al., 2005). Endothelial cells (ECs) are represented by cluster 8, which highly expresses Kdr (Hata et al., 1998). Cluster 9 highly expressed S100a8 (Laouedj et al., 2017); thus, this cluster represents immune cells (ICs). Cluster 11 represented a small group of urothelial cells (UCs), as this type of cell specifically expresses Upk3a (Kaufmann et al., 2000).

### Variable proportion of kidney cells between age-matched preterm and full-term rats

**FIGURE 3:**
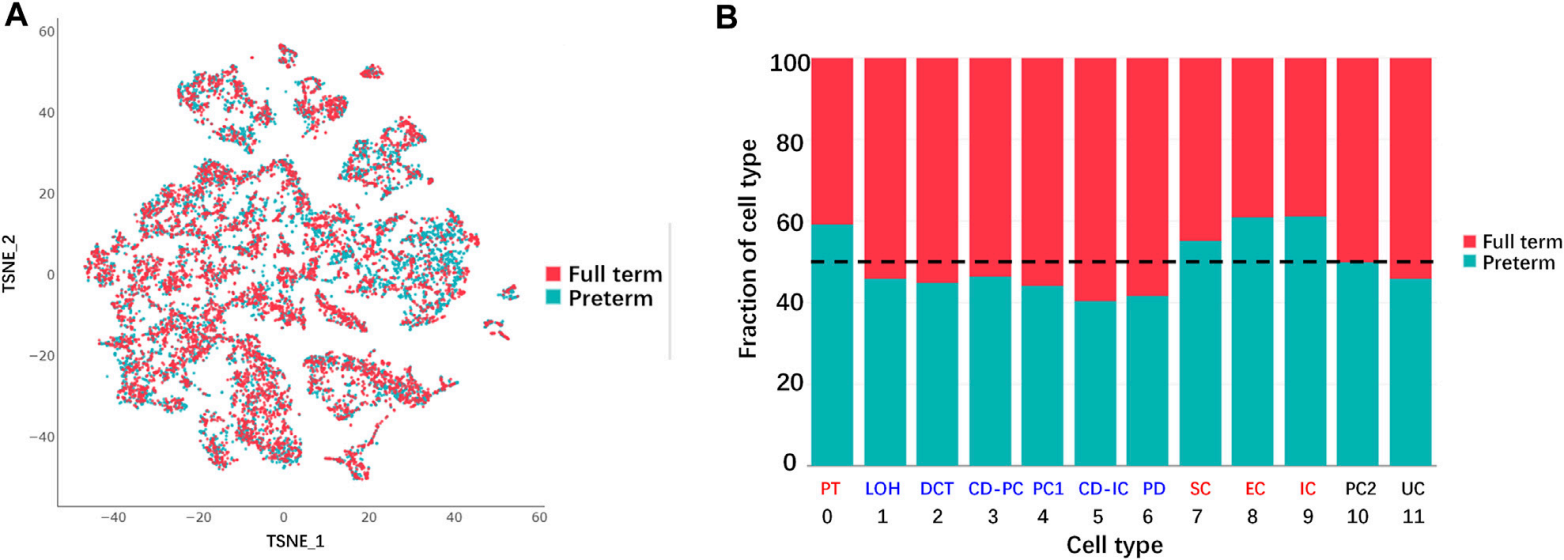
Differences in cell populations between full-term (postnatal day 0) and preterm kidneys (postnatal day 2). **(A)** Cells from full-term and preterm rat kidneys are shown in the TSNE graph. **(B)** Fraction of cells from each sample for each cluster. All samples were normalized to the same number of cells and chi square tests were used for comparison. Reduced cell numbers for the LOH, DCT, CD-PC, PC1, CD-IC, and PD populations were examined in the kidneys of preterm rats (blue color). Increasing cell numbers of PT, SC, EC and IC were detected in the kidneys of preterm rats (red color). Cells in the PC2 and UC populations were not different between full-term and preterm rat kidney (black color). (abbreviations: LOH, loop of Henle; DCT, distal convoluted tubule; CD-PC, principal cell of the collecting duct; PC1, proliferating cell 1; CD-IC, intercalated cell of the collecting duct; UC, urothelial cell; PT, proximal tubule; SC, stromal cell; EC, endothelial cell; IC, immune cell; PC2, proliferating cell 2).

For analysis of the difference in cell populations between preterm and full-term kidneys, cells from the two groups were presented in a TSNE graph (Figure 3A). The cell numbers in preterm (postnatal day 2) and full-term rats (postnatal day 0)were adjusted to be the same, and the percentages of different cell types are shown as a histogram in Figure 3B. The cell numbers of 6 cell types were decreased in the kidneys of preterm rats (postnatal day 2). These cell types were the LOH, DCT, CD-PC, PC1, CD-IC, and PD populations. The cell numbers of the PT, SC, EC and IC populations were increased in the preterm kidney. Cells in the PC2 and UC clusters were equally distributed in the two groups.

### Comparison of DEGs in the kidney between preterm and full-term rats

**FIGURE 4:**
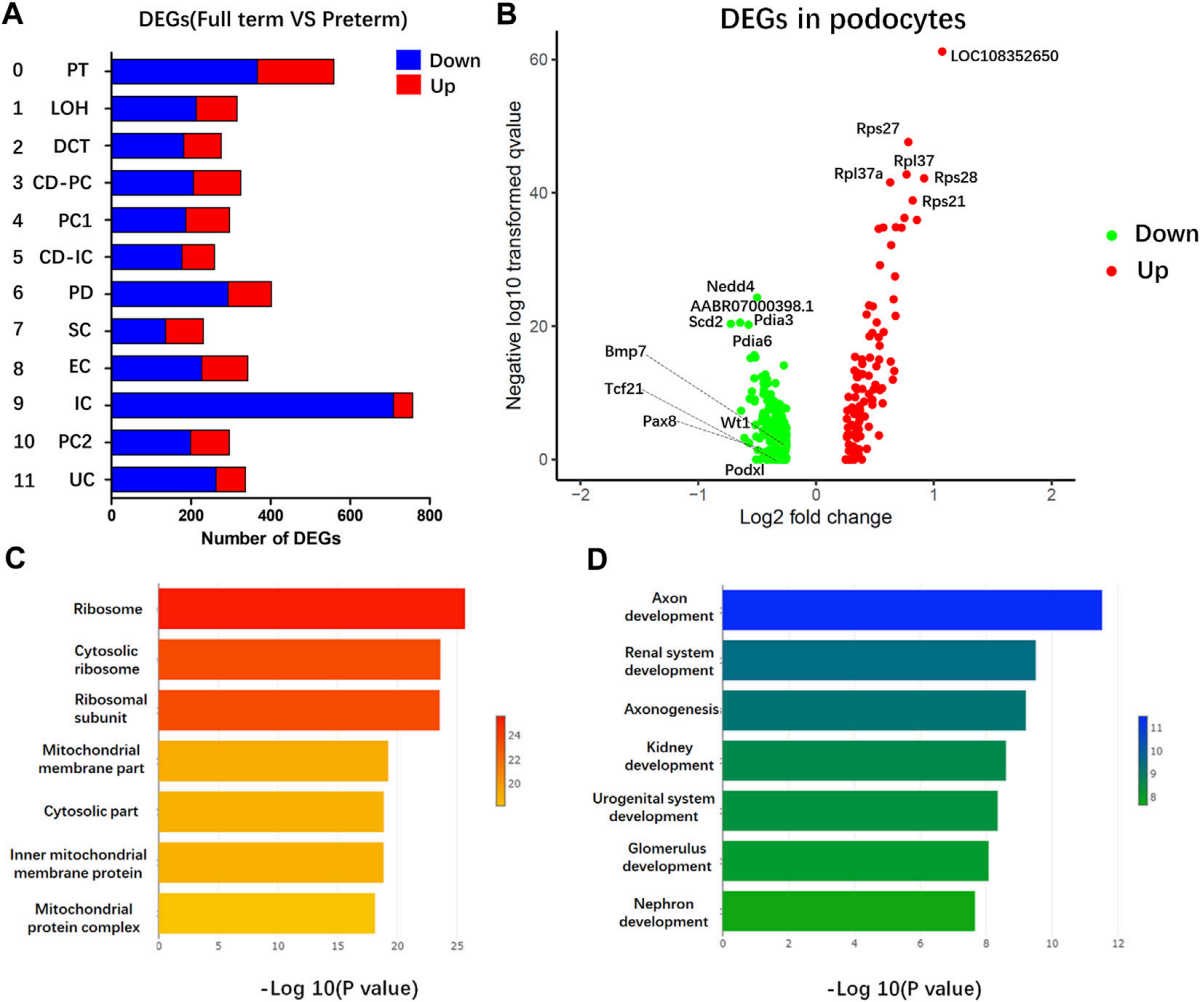
Gene expression comparison between full-term (postnatal day 0) and preterm kidneys (postnatal day 2). **(A)** Comparison between full-term and preterm kidneys according to cluster. The x-axis represents differentially expressed gene (DEG) counts between full-term and preterm kidneys. Downregulated genes are shown in blue; upregulated genes are shown in red. The y-axis represents different cell types. **(B)** Volcano plot showing DEGs in PDs. In PDs from full-term rats, downregulated genes are shown in green, and upregulated genes are shown in red. **(C,D)** GO analysis of enriched genes in PDs from full-term rats **(C)** and enriched genes in PDs from preterm rats **(D)**. In PDs from full-term rats, most functional terms are related to ribosomes. In PDs from preterm rats, most functional terms are related to kidney development.

To identify key changes in the kidneys of preterm rats (postnatal day 2), we compared data from preterm (postnatal day 2) and full-term rats (postnatal day 0) and performed differential analysis by cell type (Figure 4A). All 12 cell types showed >200 DEGs. Previous findings suggested that PDs play an important role in long-term CKD caused by preterm birth. Separate differential analysis of PDs was performed. As shown in Figure 4B, 177 genes were enriched in PDs from full-term rats (postnatal day 0), while 82 genes were enriched in PDs from preterm rats (postnatal day 2). In addition, functional GO analysis was performed. Figure 4C shows that the DEGs enriched in PDs from full-term rats (postnatal day 0) were related to ribosomes. The top DEGs were all ribosome-related genes, such as Rps27, Rps28 and Rpl37. The GO results for DEGs enriched in PDs from preterm rats (postnatal day 2) were related to renal system development (Figure 4D).

### Increased ribosome function in PDs of the full-term kidney

**FIGURE 5:**
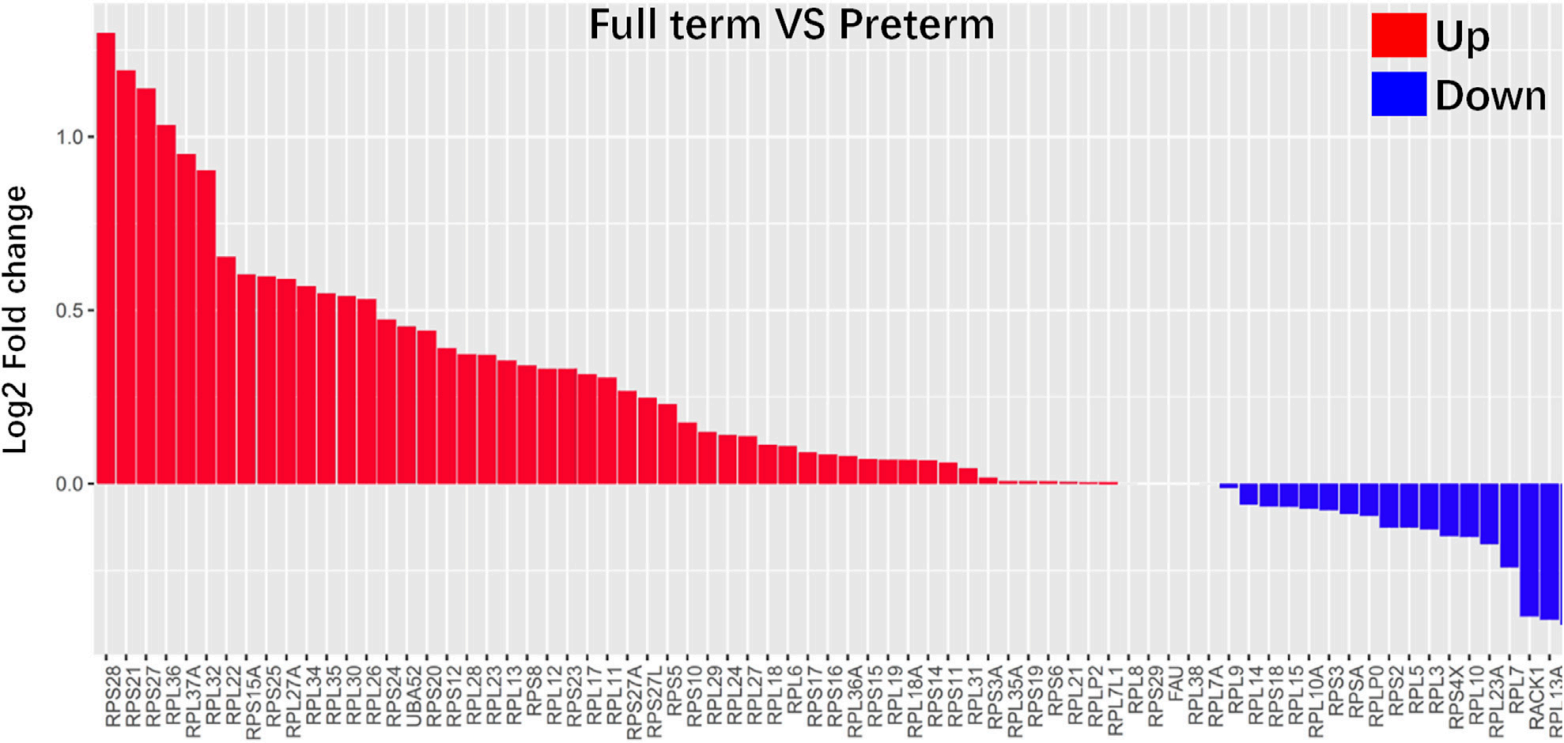
Ribosome-related genes were highly enriched in PDs from full-term rats (postnatal day 0). All 84 ribosome-related genes are listed in the Supplemental Table S2. Differences in all 84 ribosome-related genes between PDs from full-term and preterm rats were explored. The results showed that 73 of 84 ribosome-related genes were differentially expressed in PDs between preterm and full-term rats. The differences in these genes are represented as log2-fold changes. Fifty-one of 73 (approximately 70%) ribosome-related genes were enriched in PDs from full-term rats.

Functional analysis showed that ribosome-related genes were enriched in full-term rats (postnatal day 0) compared with preterm rats (postnatal day 2). As shown in Figure 5, all 84 ribosome-related genes (Supplemental Table S2) were selected, and 73 of 84 ribosome-related genes were differentially expressed in PDs between preterm and full-term rats. In addition, 51 of 73 (approximately 70%) ribosome-related genes were increased in PDs from full-term rats (postnatal day 0) compared with preterm rats (postnatal day 2).

### Accelerated maturation of PDs in the kidneys of preterm rats

**FIGURE 6:**
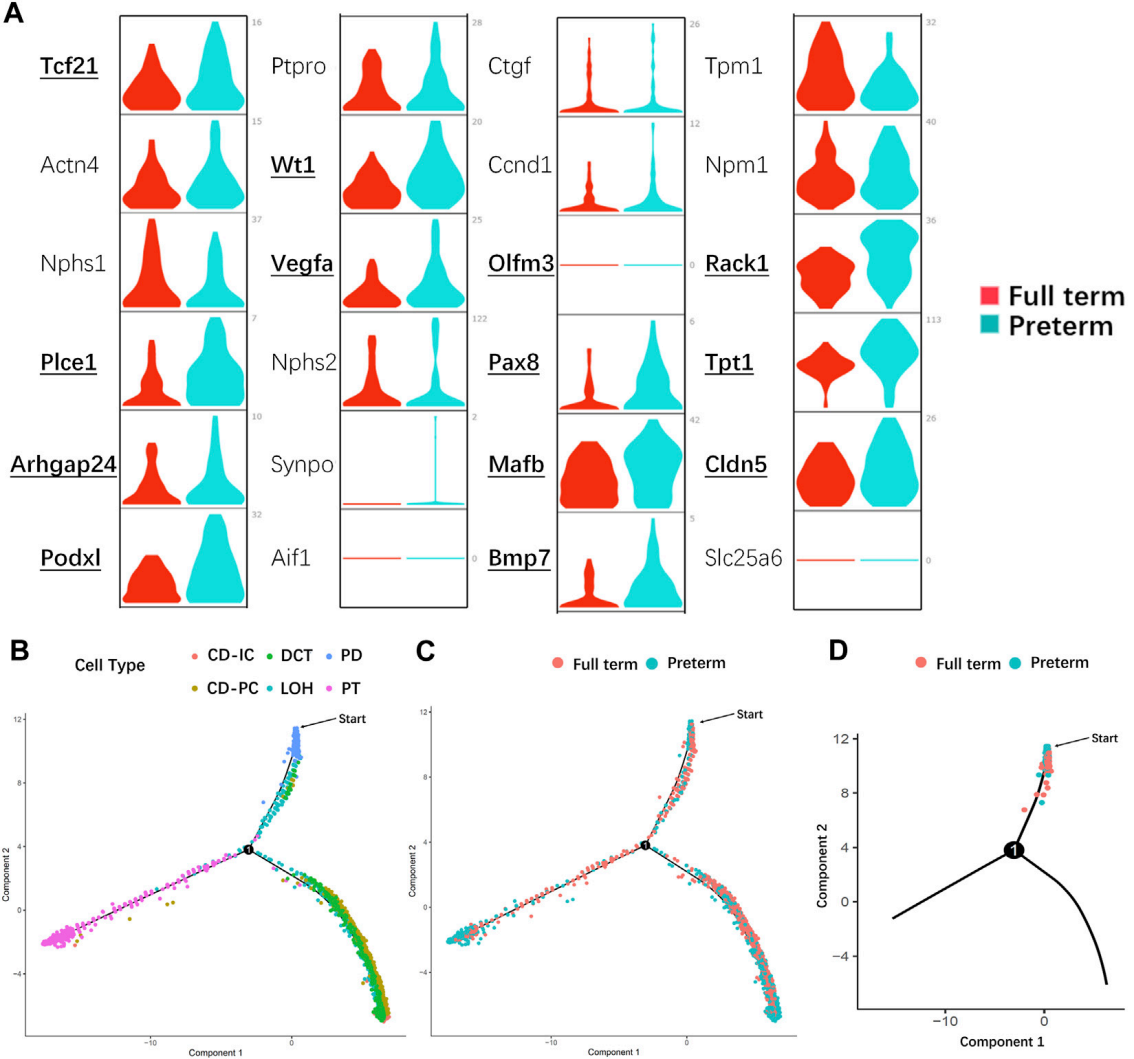
Preterm birth accelerated the maturation of PDs. **(A)** Violin plots showing the expression of known PD- and PD precursor-specific genes in the preterm (postnatal day 2) and full-term groups (postnatal day 0). X-axis: different groups, the red color represents the full-term group, and teal blue represents the preterm group; y-axis: gene expression value. Bold text indicates that there is a significant difference in that gene between the two groups. Most PD- and PD precursor-specific genes were highly expressed in PDs from preterm rats, indicating that these PDs were more mature than PDs from full-term rats. **(B)** Pseudotime trajectory of the major kidney cell types (CD-IC, CD-PC, DCT, LOH, PD, and PT). Different colors indicate different cell types. PDs (blue) were the first cell type among the progenitors to be induced. **(C)** Pseudotime trajectory of different groups of kidney cells. The red color represents the full-term group (postnatal day 0), and teal blue represents the preterm group (postnatal day 2). **(D)** In the PD split monocle pseudotime plot, PDs from preterm rats (postnatal day 2) (teal blue) were present earlier on the pseudotime path than PDs from full-term rats (postnatal day 0) (red). (Note: The starting points in **(B–D)** were applied to identify which cell population appear earlier from differentiation instead of starting of nephron progenitor cells. Abbreviations: LOH: loop of Henle, DCT: distal convoluted tubule, CD-PC: principal cell of the collecting duct, CD-IC: intercalated cell of the collecting duct, PT: proximal tubule).

Based on functional analysis, the DEGs enriched in PDs from preterm rats (postnatal day 2) (all of the DEGs listed in Supplemental Table S3) were kidney-related development (GO terms listed in Supplemental Table S4). Thus, the known PD-specific and PD precursor genes were selected and compared between the preterm (postnatal day 2) and full-term groups (postnatal day 0). As shown in Figure 6A, the expression levels of most of these genes were increased in the preterm group. This result indicated that the PDs in the preterm rat kidney were prior to maturity. To further confirm this finding, we performed pseudotemporal ordering of the major kidney cell types (PD, PT, LOH, DCT, CD-PC, and CD-IC) in the present study. As shown in Figures 6B–D, particularly the trajectories of each cell type in Figure 6B, PDs were the first cell type present. The cell trajectories of preterm and full-term rats showed that PDs were present earlier in preterm rats (postnatal day 2) than in full-term rats (postnatal day 0) (Figures 6C, D). These results indicated that preterm birth could accelerate the maturation of PDs.

## Discussion

Preterm birth is a risk factor for the future development of CKD (Hoy et al., 1999; Crump et al., 2019; Ding et al., 2021a; Gao et al., 2022). In our previous (Ding et al., 2021a) and present study, a reduction in the number of differentiated PDs was found to be involved in this process, and accelerated PD depletion was proven to be one of the causes of future CKD development. Thus, exploring the effect of preterm birth on PDs is important for clarifying the mechanism of CKD development caused by preterm birth. Based on this background, we performed scRNA-seq of kidneys collected from the same period in preterm (postnatal day 2) and full-term rats (postnatal day 0). The main findings of this study suggest that accelerated differentiation of PDs occurs in preterm individuals.

In humans, the gestational age of most preterm infants is 21–37 weeks, corresponding to the second and third trimesters (Patel et al., 2015; Stoll et al., 2015). Preterm birth interrupts the normal development of the kidney. Thus, the mechanism underlying the high risk of CKD development caused by preterm birth is closely related to abnormal development of the kidney after preterm birth. In recent years, scRNA-seq has made a massive contribution to elucidating developmental processes as well as molecular mechanisms during kidney development (Adam et al., 2017; Lindstrom et al., 2018a; Lindstrom et al., 2018b; Wang et al., 2018; Ding et al., 2021b). However, most of these studies have focused on early fetal kidney development (Adam et al., 2017; Lindstrom et al., 2018a; Lindstrom et al., 2018b; Wang et al., 2018). Few studies have focused on the middle and late stages of kidney development (Ding et al., 2021b). These two stages of kidney development correspond to the stages of abnormal kidney development in preterm individuals. In the early stage of kidney development, nephron progenitor cell dynamics and lineage specification are very important, and most studies have focused on these events (Lindstrom et al., 2018b). The fate of most cells in the kidney is determined in that stage. During middle- and late-stage development of the kidney, differentiation of the renal lineages leads to maturation, and most of the functional structures, namely, nephrons, form. The large number of functional structures formed requires a large amount of functional protein synthesis. As shown in Figures 4, 5, ribosome-related genes were enriched in the PDs of full-term rats compared to preterm rats. It is well known that ribosomes are organelles for protein synthesis (Dai and Zhu, 2020; Gabut et al., 2020; Liutkute et al., 2020). Previous studies have reported that mature cells and developing cells have different protein synthesis rate, this rate is low in mature cells but high in developing cells (Buszczak et al., 2014; Tahmasebi et al., 2018). Protein synthesis could be indicated by ribosome assembly. In present study, ribosome-related genes were enriched in the PDs of full-term rats (postnatal day 0) compared with preterm rats (postnatal day 2). Enriched ribosome-related genes indicated high rate of protein synthesis during the normal development of PDs in full term rats (postnatal day 0). In addition, previous studies also identified an increase in translation of ribosome-related mRNAs at early stages of differentiation, whereas translation of ribosome-related mRNA was inhibited at later time (Ingolia et al., 2011). This could account for PD specific genes highly expressed in preterm rat PDs. PDs were terminally differentiated cells. High expression of PD specific genes indicated that this cell population was already at the late stage of differentiation. Thus, protein synthesis rate could be low since ribosomal related genes are less abundant in preterm PDs (postnatal day 2) than its in full-term PDs (postnatal day 0). Both of these findings suggest that preterm birth could accelerate the differentiation of PDs.

As shown in Figure 4D, GO functional analysis revealed that kidney development-related genes were enriched in preterm rat PDs (postnatal day 2); these genes include PD-specific genes and some PD precursor genes, which play very important roles in PDs (Boute et al., 2000; Lindstrom et al., 2018a; Lindstrom et al., 2018b). In full-term rats, the major function of the kidney as well as PD is assisted by the maternal placenta, while preterm birth separates the kidney from the mother; thus, the preterm kidney as well as PDs begin working independently. PDs in preterm individuals must promptly adapt to the extrauterine environment and commit to kidney functions. Thus, PD-specific molecules should be appear and be enriched to meet the situation of preterm kidneys. These findings were confirmed by significantly increased expression of PD and PD precursor genes in PDs from preterm rats (postnatal day 2) (Figure 6A). In addition, based on the results of pseudotime trajectory analysis (Figures 6B–D), the appearance of PDs in preterm rats (postnatal day 2) preceded the appearance of PDs in full-term rats (postnatal day 0). Interestingly, most highly expressed genes were PD precursor specific genes. As identified by previous studies, Mafb, Cldn5, and Bmp7 are the first genes to accumulate in the PD lineage, and these genes are overall enriched in preterm PDs instead of one or in part (Lindstrom et al., 2018b; Lindstrom et al., 2018c). To deeply explore mechanism involved in this change, we should not focus on one of these genes. We should examine earlier events before appearance of PDs specific genes. The appearance of PD-specific genes suggests that terminally differentiated cells are present and that all of the differentiation process is at a late stage. Thus, this limiting factor may be events that interrupt normal overall ribosome biogenesis as well as overall protein synthesis. In present study, it is pity that we have not confirmed the detailed mechanism that regulates overall less ribosome biogenesis as well as protein synthesis in preterm PDs. This is a limitation of present study. Further studies could focus on the mechanism involved in the regulation of protein synthesis process. Nevertheless, we could also conclude that preterm birth accelerates PD differentiation. In addition, prior to the present study, Sutherland et al. reported that preterm birth could accelerate kidney maturation based on pathological examination (Sutherland et al., 2011). In that study, researchers examined kidneys from preterm neonates who survived several days. A reduced percentage of immature glomeruli was found in the preterm group compared with postconceptional age-matched gestational controls (Sutherland et al., 2011). The present study not only revealed accelerated PD differentiation but also provided evidence at the molecular level for accelerated maturation of the kidney after preterm birth.

As shown in Figure 1, preterm birth can cause a reduction in the number of differentiated mature PDs. In addition, our major findings suggested that preterm birth accelerates PD differentiation. What is the relationship between these processes? Does accelerated differentiation of PDs lead to a reduction in the final number of mature PDs? PDs are lost in both normal and diseased kidneys (Wiggins, 2007; Buszczak et al., 2014; Ding et al., 2017; Dai and Zhu, 2020). In normal kidneys, PDs are lost at a very slow speed, while PDs are excreted into the urine at a high rate in most glomerular diseases (Wiggins, 2007; Hodgin et al., 2015; Ding et al., 2017; Kikuchi et al., 2017). If we assume that the rate of PD depletion and the number of nephron progenitor cells are the same before delivery in the two groups, then after establishment of the preterm rat model by cesarean delivery 2 days early, nephron progenitor cells would immediately differentiate into PD precursors and then PDs in preterm rats. However, the overall rate of protein synthesis indicated by ribosome biogenesis could be lower in preterm PDs (postnatal day 2) than in full term PDs (postnatal day 0). Then, PD endowment could be reduced in preterm kidneys. Thus, according to our deductions, acceleration of PDs caused by preterm birth should be an intermediate factor of reduction of mature PDs instead of critical cause.

Present study showed that the developmental stage of PDs in preterm individuals is at a critical stage of protein synthesis. This process is also an important process for epigenetic modifications (Buszczak et al., 2014; Tahmasebi et al., 2018). Preterm kidney starts to independently filtrate waste as well as other kidney work after birth. Any adverse postnatal factors could affect epigenetic modification. Especially in preterm infants, different from preterm rats, preterm infants are exposed to oxygen, infection, drugs and so on. All of these adverse factors may disrupt epigenetic modifications and could lead to dysregulation of gene function without altering the DNA sequence. Both the gene and environment interactions could be determinations of final PD endowment. Further studies could also focus on epigenetic mechanisms during kidney and PD development.

In previous studies, nephron progenitor cells were identified in postnatal day 1 mouse kidney (Adam et al., 2017). However, in the present study, cluster analysis did not identify this special cell population in rat postnatal day 0 or postnatal day 2 kidneys. There were no clusters that highly expressed both classical nephron progenitor markers, such as Six2 and Cited1. As shown in Supplemental Figure S1, most of the expression features of known nephron progenitor markers have been shown (Combes et al., 2019; Naganuma et al., 2021). Interestingly, most of these nephron progenitor markers were present in cluster of PC 1 or PC2. As reported in Alexander et al., in nephron progenitor subpopulations, these marker genes in PCs were also highly expressed in the NP cell cycle cluster (Combes et al., 2019). Above all, the PC cluster may be the NPC cluster. However, PC did not differentially express the classical NPC markers Six 2 and Cited 1. Then, we still annotated these two clusters as proliferating cells.

Our study focused on PDs, and we did not analyze other inherent kidney cell types. However, as shown in Figure 2, except for PT cells, the numbers of most types of kidney cells in preterm rats were smaller than those in full-term rats. Except for PC cells, most types of cells are major constituents of nephrons. The decreased number of these cells suggested a smaller number of nephrons in preterm rats. This finding confirmed the nephron number hypothesis. Although we have not thoroughly explored pattern changes in other types of kidney cells, the present study could provide a resource for researchers to achieve a deep understanding of molecular and regulatory events in preterm and full-term kidneys.

## Data Availability

The data presented in the study are deposited in the Sequence Read Archive repository, accession number PRJNA861698.
