# Supplementary figures and images for: Preterm birth leads to a decreased number of differentiated podocytes and accelerated podocyte differentiation
Source: Front Cell Dev Biol. 2023 Mar 2;11:1142929. doi: 10.3389/fcell.2023.1142929 (PMC10018169; doi:10.3389/fcell.2023.1142929)

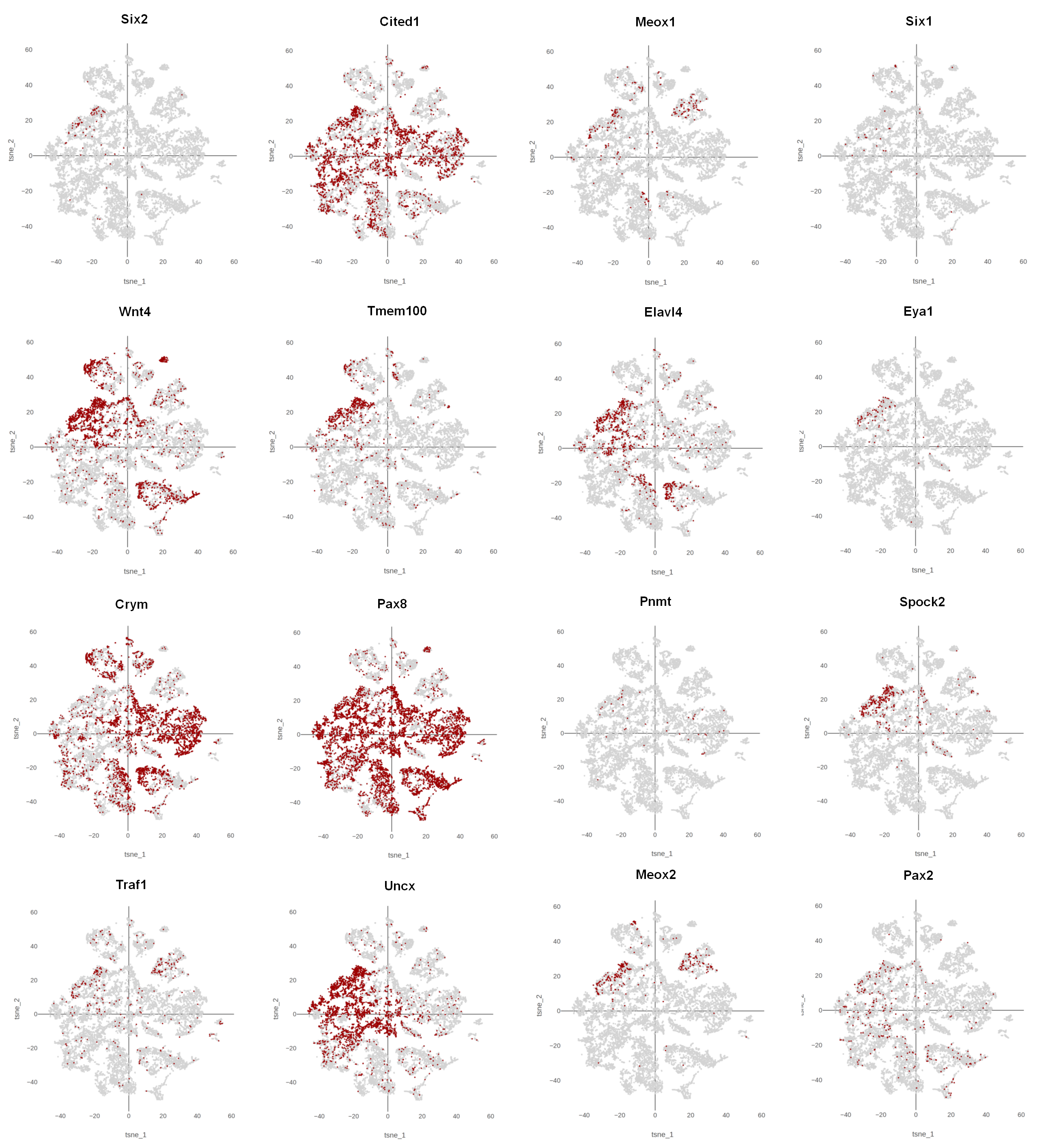

Supplement: Supplementary file 3 [file Image1.TIF]
