# Supplementary material for: Preterm birth leads to a decreased number of differentiated podocytes and accelerated podocyte differentiation
Source: Front Cell Dev Biol. 2023 Mar 2;11:1142929. doi: 10.3389/fcell.2023.1142929 (PMC10018169; doi:10.3389/fcell.2023.1142929)
Supplement: Supplementary file 6 [file DataSheet1.docx]

**Supplementary information summary**

Supplemental Table 1. Median expression levels of each gene in original 20 clusters.

Supplemental Table 2. List of all of 84 ribosome related genes.

Supplemental Table 3. List of differentially expressed genes between full term and preterm podocytes.

Supplemental Table 4. GO terms (Biological Process) based on differentially expressed genes enriched in preterm podocytes.

Supplemental Figure 1. Expressions pattern of previous known nephron progenitor marker genes in TSNE plots.
